# Supplementary material for: Factors associated with interstitial lung disease in patients with rheumatoid arthritis: A systematic review and meta-analysis
Source: PLoS One. 2023 Jun 23;18(6):e0286191. doi: 10.1371/journal.pone.0286191 (PMC10289414; doi:10.1371/journal.pone.0286191)
Supplement: S1 Table — (DOC) [file pone.0286191.s001.doc]

**Table 1 Characteristics of the included studies**

| **Author** | **Year** | **Study design**  **(Quality score)** | **RA Classification criteria** | **RA-ILD** | | | | | **RA without ILD** | | | |
| --- | --- | --- | --- | --- | --- | --- | --- | --- | --- | --- | --- | --- |
|  |  |  |  | **Subjects (*n*)**  **(M/F)** | **Average age (years)^a^** | **Duration of RA (years)^a^** | **Age at onset of RA (years)^a^** | **ILD diagnosis methods** | **Subjects (*n*)**  **(M/F)** | **Average age (years)^a^** | **Duration of RA (years)^a^** | **Age at onset of RA (years)^a^** |
| Ben [8] | 2022 | Retrospective cohort (7) | ACR/EULAR 2010 criteria | 52 (14/38) | 66.3 ±11 | 13.3 ±7.8 | 47.2 ±13.5 | A, B, D HRCT | 52 (14/38) | 65.6 ±10.8 | 12.2 ±7.49 | 48.0 ±12.8 |
| Denis [9] | 2022 | Retrospective cohort (8) | ACR/EULAR 2010 criteria | 89 (41/48) | 71 (66–76) | NA | NA | HRCT | 1411 (374/1037) | 65 (55–74) | NA | NA |
| Ong [10] | 2022 | Retrospective cohort (7) | ACR/EULAR 2010 criteria | 54 (3/51) | 59.6 ±11.9 | 7 (7) | 50.3 ±13.0 | HRCT | 678 (106/572) | 58.0 ±13.5 | 8 (8) | 48.9 ±13.4 |
| Severo [11] | 2022 | Cross-sectional (10) | ACR/EULAR 2010 criteria | 49 (10/39) | 61 ± 11 | 13 ± 9 | NA | D and CT | 85 (4/81) | 61 ± 11 | 13 ± 9 | NA |
| Kronzer [12] | 2021 | Case-control (8) | 1987 ACR or ACR/EULAR 2010 criteria | 84 (19/65) | 67 ±10 | 20 ±12 | NA | HRCT | 233 (47/186) | 66 ±11 | 20 ±11 | NA |
| Wickrematilake [13] | 2021 | Retrospective cohort (7) | 1987 ACR or ACR/EULAR 2010 criteria | 56 (9/47) | 52.9 | 7.7 | NA | B and HRCT | 328 (37/291) | NA | NA | NA |
| Del [14] | 2020 | Case-control (7) | ACR/EULAR 2010 criteria and ATS/ERS 2002 criteria | 65(12/53) | 61 (37–85) | NA | 53 (23–85) | HRCT | 82(1/81) | 53.5 (25–80) | NA | 45.5 (18–75) |
| Li [15] | 2020 | Retrospective cohort (6) | 1987 ACR or ACR/EULAR 2010 criteria | 278 (108/170) | 52.6 ±15.0 | 36 (10, 118) (months) | 57.4 ±13.9 | HRCT | 645 (145/500) | 52.6 ±15.0 | 86 (36, 184) (months) | 50.6 ± 15.0 |
| Wang [16] | 2020 | Retrospective cohort (7) | ACR/EULAR 2010 criteria | 45 (18/27) | 59.5 ±8.7 | 7.2 ±6.2 | 54.0 ±12.2 | A, B and HRCT | 51 (14/37) | 46.1±12.2 | 6.0 ±4.2 | 40.4 ±11.7 |
| Lai [17] | 2019 | Case-control (7) | 1987 ACR criteria | 100 (43/57) | 63.9 ±9.0 | 9.9 ±10.2 | NA | NA | 100 (25/75) | 53.3 ±13.2 | 9.1 ±8.3 | NA |
| Qin [18]^b^ | 2019 | Case-control (6) | 1987 ACR or ACR/EULAR 2010 criteria | 229 (65/164) | 61.2 ±10.5 | 72 (24, 120) (months) | 52.8 ±13.8 | HRCT | 144 (22/122) | 54.6 ±12.5 | 60 (12, 120) (months) | 47.5 ±13.7 |
| Salaffi [19] | 2019 | Retrospective cohort (7) | 2010 ACR criteria | 29 (NA) | 66.6 ±10.3 | 7.5 ±2.8 | 59.4 ±9.4 | D, E and HRCT | 122 (NA) | 54.7 ±7.8 | 7.5 ±4.0 | 46.0 ±7.9 |
| Yang [20] | 2019 | Retrospective cohort (7) | 1987 ACR criteria | 77 (19/58) | 56.6 ±13.1 | 11.5 ±8.5 | NA | A, D, F and/or C | 231 (57/174) | 57.1 ±11.7 | 10.8 ±6.9 | NA |
| Sacks [21] | 2018 | Cross sectional (9) | ACR/EULAR 2010 criteria | 63 (8/55) | 50.0 ±9.2 | 10.1 ±60.1 | NA | HRCT | 25 (5/20) | 48.4 ±8.2 | 10.4 ±6.5 | NA |
| Zhang [22] | 2017 | Retrospective cohort (6) | 1987 ACR or ACR/EULAR 2010 criteria | 237 (86/151) | 57.6 ±13.2 | 24 (months) | NA | HRCT | 313 (79/234) | 47.7±14.5 | 48 (months) | NA |
| Chen [23]^b^ | 2016 | Retrospective cohort (7) | 1987 ACR criteria | 35 (16/19) | 62.4 ±2.3 | 6.3 | 56.1 ±2.6 | HRCT | 82  (16 /66) | 52.6 ±1.4 | 8.9 | 43.6 ±1.6 |
| Restrepo [24] | 2015 | Cross sectional (8) | 1987 ACR criteria | 69 (34/35) | 60.2 ±9.7 | 12.6 ±10.8 | 47.4 ±12.6 | B, CT, HRCT, C | 563 (127/436) | 52.9 ±13.5 | 10.2 ±10.2 | 42.6 ±13.9 |
| Wang [25] | 2015 | Retrospective cohort (7) | 2006 ACR criteria | 83 (29/54) | 59.6 ±9.7 | 7.5 ±7.4 | 52.1 ±11.9 | HRCT | 461 (88/373) | 50.5 ±13.8 | 5.3 ±6.3 | 45.3 ±14.2 |
| Yin [26] | 2014 | Retrospective cohort (5) | 1987 ACR criteria | 71 (21/50) | 58.3 ±11.2 | 9.0 (2.0–18.0) | NA | HRCT | 214 (53/161) | 49.5 ±13.4 | 4.0 (1.0–10.1) | NA |
| Mori [27] | 2012 | Retrospective cohort (6) | 1987 ACR criteria | 24 (12/12) | 72.5 (64.0, 76.3) | 1.5 (0, 6.3) | NA | HRCT | 302 (70/232) | 59.0 (52.0, 68.0) | 0 (0, 6.0) | NA |
| Bongartz [28] | 2010 | Retrospective cohort (7) | 1987 ACR criteria | 46 (27/19) | NA | NA | 56.8 ±14.7 | F, CT, A, D, C | 536 (128/408) | NA | NA | 57.8 ±15.2 |
| Koduri [29] | 2010 | Retrospective cohort (6) | ACR criteria | 52 (22/30) | NA | 7 (5, 11) | 65 (58, 71) | A, B, HRCT | 1408 (469/ 939) | NA | 6 (4, 11) | 56 (45, 66) |

Abbreviations: RA, rheumatoid arthritis; RA-ILD, rheumatoid arthritis-associated interstitial lung disease; M/F, Male/Female; ACR, American College of Rheumatology; EULAR, the European League Against Rheumatism; A, clinical symptoms; B, chest x-ray; C, lung biopsy; D, pulmonary function tests (PFTs); HRCT, High-resolution chest computed tomography; NA, not available; CT, computed tomography; ATS/ERS, American Thoracic Society/European Respiratory Society; E, dyspnea scale; F, radiographic evidence.

a: Values are presented as median (range), Median (IQR) or mean ± SD.

b: Articles written in Chinese.
