# Supplementary material for: Factors associated with interstitial lung disease in patients with rheumatoid arthritis: A systematic review and meta-analysis
Source: PLoS One. 2023 Jun 23;18(6):e0286191. doi: 10.1371/journal.pone.0286191 (PMC10289414; doi:10.1371/journal.pone.0286191)
Supplement: S2 Table — (DOC) [file pone.0286191.s002.doc]

Table 2. Quality scores of cross-sectional studies using AHRQ.

| Author and year | Q1 | Q2 | Q3 | Q4 | Q5 | Q6 | Q7 | Q8 | Q9 | Q10 | Q11 | Score |
| --- | --- | --- | --- | --- | --- | --- | --- | --- | --- | --- | --- | --- |
| Severo [11] | 1 | 1 | 1 | 0 | 0 | 2 | 1 | 1 | 0 | 0 | 0 | 7 |
| Sacks [21] | 1 | 1 | 1 | 0 | 0 | 0 | 1 | 1 | 0 | 0 | 0 | 5 |
| Restrepo [24] | 1 | 1 | 1 | 0 | 0 | 2 | 1 | 1 | 0 | 0 | 0 | 7 |

Notes: Q1. Define the source of information (survey, record review); Q2. List inclusion and exclusion criteria for exposed and unexposed subjects (cases and controls) or refer to previous publications; Q3. Indicate time period used for identifying patients; Q4. Indicate whether or not subjects were consecutive if not population-based; Q5. Indicate if evaluators of subjective components of study were masked to other aspects of the status of the participants; Q6. Describe any assessments undertaken for quality assurance purposes (e.g., test/retest of primary outcome measurements); Q7. Explain any patient exclusions from analysis; Q8. Describe how confounding was assessed and/or controlled; Q9. If applicable, explain how missing data were handled in the analysis; Q10. Summarize patient response rates and completeness of data collection; Q11. Clarify what follow-up, if any, was expected and the percentage of patients for which incomplete data or follow-up was obtained.

Table 3. Quality scores of cohort or case-control studies using Newcastle-Ottawa Scale.

| Study | Selection | | | | Comparability | Outcome | | | NOS |
| --- | --- | --- | --- | --- | --- | --- | --- | --- | --- |
|  | Q1 | Q2 | Q3 | Q4 | Q5 | Q6 | Q7 | Q8 | Overall score |
| Ben [8] | 1 | 1 | 1 | 1 | 1 | 1 | 1 | 0 | 7 |
| Denis [9] | 1 | 1 | 1 | 1 | 2 | 1 | 1 | 0 | 8 |
| Ong [10] | 1 | 1 | 1 | 1 | 1 | 1 | 1 | 0 | 7 |
| Kronzer [12] | 1 | 1 | 1 | 1 | 2 | 1 | 1 | 0 | 8 |
| Wickrematilake [13] | 1 | 1 | 1 | 1 | 1 | 1 | 1 | 0 | 7 |
| Del [14] | 1 | 1 | 1 | 1 | 1 | 1 | 1 | 0 | 7 |
| Li [15] | 0 | 1 | 1 | 1 | 1 | 1 | 1 | 0 | 6 |
| Wang [16] | 1 | 1 | 1 | 1 | 1 | 1 | 1 | 0 | 7 |
| Lai [17] | 1 | 1 | 1 | 1 | 1 | 1 | 1 | 0 | 7 |
| Qin [18] | 0 | 1 | 1 | 1 | 1 | 1 | 1 | 0 | 6 |
| Salaffi [19] | 1 | 1 | 1 | 1 | 1 | 1 | 1 | 0 | 7 |
| Yang [20] | 1 | 1 | 1 | 1 | 1 | 1 | 1 | 0 | 7 |
| Zhang [22] | 0 | 1 | 1 | 1 | 1 | 1 | 1 | 0 | 6 |
| Chen [23] | 1 | 1 | 1 | 1 | 1 | 1 | 1 | 0 | 7 |
| Wang [25] | 1 | 1 | 1 | 1 | 1 | 1 | 1 | 0 | 7 |
| Yin [26] | 0 | 0 | 1 | 1 | 1 | 1 | 1 | 0 | 5 |
| Mori [27] | 0 | 1 | 1 | 1 | 1 | 1 | 1 | 0 | 6 |
| Bongartz [28] | 1 | 1 | 1 | 1 | 1 | 1 | 1 | 0 | 7 |
| Koduri [29] | 0 | 1 | 1 | 1 | 1 | 1 | 1 | 0 | 6 |

Notes: Q1. Representativeness of the exposed cohort; Q2. Selection of the non exposed cohort; Q3. Ascertainment of exposure; Q4. Demonstration that outcomes was not present at start of study; Q5. Comparability on the basis of the design or analysis; Q6. Assessment of outcome; Q7. Adequate follow-up duration; Q8. Adequate follow-up rate
